# Supplementary material for: OpenMM 7: Rapid development of high performance algorithms for molecular dynamics
Source: PLoS Comput Biol. 2017 Jul 26;13(7):e1005659. doi: 10.1371/journal.pcbi.1005659 (PMC5549999; doi:10.1371/journal.pcbi.1005659)
Supplement: S1 Source Code — (ZIP) [file pcbi.1005659.s002.zip › OpenMM-7.0.1-Source/docs/api-c++/generated/OpenMM.System.html]

System — OpenMM @OPENMM\_MAJOR\_VERSION@.@OPENMM\_MINOR\_VERSION@ documentation


# System¶

*class* `OpenMM::``System`¶
:   This class represents a molecular system. The definition of a `System` involves four elements:

    > - The set of particles in the system
    > - The forces acting on them
    > - Pairs of particles whose separation should be constrained to a fixed value
    > - For periodic systems, the dimensions of the periodic box

    The particles and constraints are defined directly by the `System` object, while forces are defined by objects that extend the `Force` class. After creating a `System`, call `addParticle()` once for each particle, `addConstraint()` for each constraint, and `addForce()` for each `Force`.

    In addition, particles may be designated as “virtual sites”. These are particles whose positions are computed automatically based on the positions of other particles. To define a virtual site, call `setVirtualSite()`, passing in a `VirtualSite` object that defines the rules for computing its position.

    Methods

    |  |  |
    | --- | --- |
    | `System` | Create a new `System`. |
    | `~System` |  |
    | `getNumParticles` | Get the number of particles in this `System`. |
    | `addParticle` | Add a particle to the `System`. |
    | `getParticleMass` | Get the mass (in atomic mass units) of a particle. |
    | `setParticleMass` | Set the mass (in atomic mass units) of a particle. |
    | `setVirtualSite` | Set a particle to be a virtual site. |
    | `isVirtualSite` | Get whether a particle is a `VirtualSite`. |
    | `getVirtualSite` | Get `VirtualSite` object for a particle. |
    | `getNumConstraints` | Get the number of distance constraints in this `System`. |
    | `addConstraint` | Add a constraint to the `System`. |
    | `getConstraintParameters` | Get the parameters defining a distance constraint. |
    | `setConstraintParameters` | Set the parameters defining a distance constraint. |
    | `removeConstraint` | Remove a constraint from the `System`. |
    | `addForce` | Add a `Force` to the `System`. |
    | `getNumForces` | Get the number of `Force` objects that have been added to the `System`. |
    | `getForce` | Get a const reference to one of the Forces in this `System`. |
    | `getForce` | Get a writable reference to one of the Forces in this `System`. |
    | `removeForce` | Remove a `Force` from the `System`. |
    | `getDefaultPeriodicBoxVectors` | Get the default values of the vectors defining the axes of the periodic box (measured in nm). |
    | `setDefaultPeriodicBoxVectors` | Set the default values of the vectors defining the axes of the periodic box (measured in nm). |
    | `usesPeriodicBoundaryConditions` | Returns whether or not any forces in this `System` use periodic boundaries. |

    `System`()¶
    :   Create a new `System`.

    `~System`()¶

    int `getNumParticles`() *const*¶
    :   Get the number of particles in this `System`.

    int `addParticle`(double *mass*)¶
    :   Add a particle to the `System`. If the mass is 0, Integrators will ignore the particle and not modify its position or velocity. This is most often used for virtual sites, but can also be used as a way to prevent a particle from moving.

        |  |  |
        | --- | --- |
        | Parameters: |  |

        - **mass** – the mass of the particle (in atomic mass units)

        |  |  |
        | --- | --- |
        | Returns: | the index of the particle that was added |

    double `getParticleMass`(int *index*) *const*¶
    :   Get the mass (in atomic mass units) of a particle. If the mass is 0, Integrators will ignore the particle and not modify its position or velocity. This is most often used for virtual sites, but can also be used as a way to prevent a particle from moving.

        |  |  |
        | --- | --- |
        | Parameters: |  |

        - **index** – the index of the particle for which to get the mass

    void `setParticleMass`(int *index*, double *mass*)¶
    :   Set the mass (in atomic mass units) of a particle. If the mass is 0, Integrators will ignore the particle and not modify its position or velocity. This is most often used for virtual sites, but can also be used as a way to prevent a particle from moving.

        |  |  |
        | --- | --- |
        | Parameters: |  |

        - **index** – the index of the particle for which to set the mass
        - **mass** – the mass of the particle

    void `setVirtualSite`(int *index*, VirtualSite \**virtualSite*)¶
    :   Set a particle to be a virtual site. The `VirtualSite` object should have been created on the heap with the “new” operator. The `System` takes over ownership of it, and deletes it when the `System` itself is deleted.

        |  |  |
        | --- | --- |
        | Parameters: |  |

        - **index** – the index of the particle that should be treated as a virtual site
        - **virtualSite** – a pointer to the `VirtualSite` object describing it

    bool `isVirtualSite`(int *index*) *const*¶
    :   Get whether a particle is a `VirtualSite`.

        |  |  |
        | --- | --- |
        | Parameters: |  |

        - **index** – the index of the particle to check

    *const* VirtualSite &`getVirtualSite`(int *index*) *const*¶
    :   Get `VirtualSite` object for a particle. If the particle is not a virtual site, this throws an exception.

        |  |  |
        | --- | --- |
        | Parameters: |  |

        - **index** – the index of the particle to get

    int `getNumConstraints`() *const*¶
    :   Get the number of distance constraints in this `System`.

    int `addConstraint`(int *particle1*, int *particle2*, double *distance*)¶
    :   Add a constraint to the `System`. Particles whose mass is 0 cannot participate in constraints.

        |  |  |
        | --- | --- |
        | Parameters: |  |

        - **particle1** – the index of the first particle involved in the constraint
        - **particle2** – the index of the second particle involved in the constraint
        - **distance** – the required distance between the two particles, measured in nm

        |  |  |
        | --- | --- |
        | Returns: | the index of the constraint that was added |

    void `getConstraintParameters`(int *index*, int &*particle1*, int &*particle2*, double &*distance*) *const*¶
    :   Get the parameters defining a distance constraint.

        |  |  |
        | --- | --- |
        | Parameters: |  |

        - **index** – the index of the constraint for which to get parameters
        - **particle1** – [out] the index of the first particle involved in the constraint
        - **particle2** – [out] the index of the second particle involved in the constraint
        - **distance** – [out] the required distance between the two particles, measured in nm

    void `setConstraintParameters`(int *index*, int *particle1*, int *particle2*, double *distance*)¶
    :   Set the parameters defining a distance constraint. Particles whose mass is 0 cannot participate in constraints.

        |  |  |
        | --- | --- |
        | Parameters: |  |

        - **index** – the index of the constraint for which to set parameters
        - **particle1** – the index of the first particle involved in the constraint
        - **particle2** – the index of the second particle involved in the constraint
        - **distance** – the required distance between the two particles, measured in nm

    void `removeConstraint`(int *index*)¶
    :   Remove a constraint from the `System`.

        |  |  |
        | --- | --- |
        | Parameters: |  |

        - **index** – the index of the constraint to remove

    int `addForce`(Force \**force*)¶
    :   Add a `Force` to the `System`. The `Force` should have been created on the heap with the “new” operator. The `System` takes over ownership of it, and deletes the `Force` when the `System` itself is deleted.

        |  |  |
        | --- | --- |
        | Parameters: |  |

        - **force** – a pointer to the `Force` object to be added

        |  |  |
        | --- | --- |
        | Returns: | the index within the `System` of the `Force` that was added |

    int `getNumForces`() *const*¶
    :   Get the number of `Force` objects that have been added to the `System`.

    *const* Force &`getForce`(int *index*) *const*¶
    :   Get a const reference to one of the Forces in this `System`.

        |  |  |
        | --- | --- |
        | Parameters: |  |

        - **index** – the index of the `Force` to get

    Force &`getForce`(int *index*)¶
    :   Get a writable reference to one of the Forces in this `System`.

        |  |  |
        | --- | --- |
        | Parameters: |  |

        - **index** – the index of the `Force` to get

    void `removeForce`(int *index*)¶
    :   Remove a `Force` from the `System`. The memory associated with the removed `Force` object is deleted.

        |  |  |
        | --- | --- |
        | Parameters: |  |

        - **index** – the index of the `Force` to remove

    void `getDefaultPeriodicBoxVectors`(Vec3 &*a*, Vec3 &*b*, Vec3 &*c*) *const*¶
    :   Get the default values of the vectors defining the axes of the periodic box (measured in nm). Any newly created `Context` will have its box vectors set to these. They will affect any `Force` added to the `System` that uses periodic boundary conditions.

        |  |  |
        | --- | --- |
        | Parameters: |  |

        - **a** – [out] the vector defining the first edge of the periodic box
        - **b** – [out] the vector defining the second edge of the periodic box
        - **c** – [out] the vector defining the third edge of the periodic box

    void `setDefaultPeriodicBoxVectors`(*const* Vec3 &*a*, *const* Vec3 &*b*, *const* Vec3 &*c*)¶
    :   Set the default values of the vectors defining the axes of the periodic box (measured in nm). Any newly created `Context` will have its box vectors set to these. They will affect any `Force` added to the `System` that uses periodic boundary conditions.

        Triclinic boxes are supported, but the vectors must satisfy certain requirements. In particular, a must point in the x direction, b must point “mostly” in the y direction, and c must point “mostly” in the z direction. See the documentation for details.

        |  |  |
        | --- | --- |
        | Parameters: |  |

        - **a** – the vector defining the first edge of the periodic box
        - **b** – the vector defining the second edge of the periodic box
        - **c** – the vector defining the third edge of the periodic box

    bool `usesPeriodicBoundaryConditions`() *const*¶
    :   Returns whether or not any forces in this `System` use periodic boundaries.

        If a force in this `System` does not implement usesPeriodicBoundaryConditions a `OpenMM::OpenMMException` is thrown

        |  |  |
        | --- | --- |
        | Returns: | true if at least one force uses PBC and false otherwise |

High performance molecular simulation on GPUs


### Navigation

- Core Objects
- Forces
- Integrators
- Extras

©2015, Stanford University and the Authors.
|
Powered by Sphinx 1.3.1
& Alabaster 0.7.7
|
Page source
